# An Improved Mutual Information Feature Selection Technique for Intrusion Detection Systems in the Internet of Medical Things

**DOI:** 10.3390/s23104971

**Published:** 2023-05-22

**Authors:** Mousa Alalhareth, Sung-Chul Hong

**Affiliations:** 1 Department of Information Systems, College of Computer Science and Information System, Najran University, Najran 61441, Saudi Arabia; 2 Department of Computer and Information Sciences, Towson University, Towson, MD 21204, USA

**Keywords:** IDS, IoMT, LRGU, MIFS, feature selection, machine learning, deep learning

## Abstract

In healthcare, the Internet of Things (IoT) is used to remotely monitor patients and provide real-time diagnoses, which is referred to as the Internet of Medical Things (IoMT). This integration poses a risk from cybersecurity threats that can harm patient data and well-being. Hackers can manipulate biometric data from biosensors or disrupt the IoMT system, which is a major concern. To address this issue, intrusion detection systems (IDS) have been proposed, particularly using deep learning algorithms. However, developing IDS for IoMT is challenging due to high data dimensionality leading to model overfitting and degraded detection accuracy. Feature selection has been proposed to prevent overfitting, but the existing methods assume that feature redundancy increases linearly with the size of the selected features. Such an assumption does not hold, as the amount of information a feature carries about the attack pattern varies from feature to feature, especially when dealing with early patterns, due to data sparsity that makes it difficult to perceive the common characteristics of selected features. This negatively affects the ability of the mutual information feature selection (MIFS) goal function to estimate the redundancy coefficient accurately. To overcome this issue, this paper proposes an enhanced feature selection technique called Logistic Redundancy Coefficient Gradual Upweighting MIFS (LRGU-MIFS) that evaluates candidate features individually instead of comparing them with common characteristics of the already-selected features. Unlike the existing feature selection techniques, LRGU calculates the redundancy score of a feature using the logistic function. It increases the redundancy value based on the logistic curve, which reflects the nonlinearity of the relationship of the mutual information between features in the selected set. Then, the LRGU was incorporated into the goal function of MIFS as a redundancy coefficient. The experimental evaluation shows that the proposed LRGU was able to identify a compact set of significant features that outperformed those selected by the existing techniques. The proposed technique overcomes the challenge of perceiving common characteristics in cases of insufficient attack patterns and outperforms existing techniques in identifying significant features.

## 1. Introduction

The Internet of Medical Things (IoMT) refers to the network of medical devices and equipment connected to the internet, allowing for the exchange of data and information [1,2]. IoMT includes a wide range of devices, such as wearable devices, remote monitoring systems, and in-home diagnostic devices, which can be used to collect and transmit patient data to healthcare providers [3]. The use of IoMT has the potential to transform the healthcare industry by improving patient outcomes, reducing costs, and increasing efficiency. IoMT devices can provide a continuous monitoring of patient health, allowing healthcare providers to intervene early and prevent serious health problems from developing [4]. Additionally, IoMT devices can also help to reduce the burden on healthcare systems by enabling patients to receive care in their own homes, reducing the need for hospitalization and increasing access to care [5]. Despite the potential benefits of IoMT, it is important to ensure the security and privacy of patient data, as well as the reliability of the devices, to avoid potential harm to patients and to maintain public trust in the healthcare system [6].

The data generated by the IoMT is distinct from that of other IoT applications due to its health-centric nature, high sensitivity, and the stringent privacy and accuracy requirements it demands [7]. IoMT data is primarily health-related, encompassing several medical and health parameters representing patient vital signs [1]. Its complexity and sensitivity require stringent privacy and security measures, in compliance with regulations such as the Health Insurance Portability and Accountability Act (HIPAA) [8]. In addition, IoMT data often demand higher data integrity and accuracy, as it directly influences clinical decision-making and patient outcomes, contrasting with some IoT applications where occasional inaccuracies can be tolerated, as they may not carry severe consequences. Moreover, the temporal aspects of data also vary.

IoMT data are also characterized by continuous, real-time data streams, enabling constant health monitoring, in contrast to the potentially sporadic nature of IoT data [7]. Furthermore, IoMT data exhibit a higher degree of interdependence and correlation between attributes, reflecting the interconnected nature of physiological variables [9]. For example, heart rate and blood pressure may be highly correlated, since both can increase during periods of stress. Recognizing such correlations can help feature selection, possibly allowing the removal of one feature to reduce model complexity without significantly impacting performance. This interdependence can be leveraged for feature selection in data preprocessing, as understanding these correlations can help identify redundant or irrelevant features, reducing data dimensionality and improving model performance. 

The IoMT is vulnerable to a range of cyber threats that can compromise patient safety and privacy, as well as the reliability of the devices [10,11]. These threats include data breaches, unauthorized access to patient information, tampering with medical devices, and malware infections [12,13]. In particular, the use of IoMT devices in healthcare environments introduces new attack surfaces and vulnerabilities that can be exploited by malicious actors [14,15]. For example, the lack of security measures on some IoMT devices can allow unauthorized access to sensitive patient data, while malware infections can compromise the functionality of the devices and disrupt the delivery of care [6,16]. Additionally, the use of IoMT devices in critical care settings, such as operating rooms or intensive care units, can have serious consequences if the devices are compromised [17]. To mitigate these threats, it is important to implement robust security measures, such as encryption and authentication, to protect patient data and the devices themselves [18]. Additionally, healthcare organizations should implement regular security updates and monitoring to detect and respond to potential cyber threats in a timely manner [14].

Several solutions have been proposed to countermeasure the cyber threats faced by IoMT devices [19]. One approach is to implement secure protocols and encryption mechanisms to protect the data transmitted between IoMT devices and healthcare systems [19]. This can include the use of secure communication standards, such as a secure socket layer (SSL) and transport layer security (TLS), to ensure that data are transmitted securely and cannot be tampered with or intercepted [20,21,22]. Another solution is to improve the security of the devices themselves, for example, by implementing secure boot mechanisms to prevent malware infections and ensuring that software updates are securely transmitted and installed [23,24]. In addition, researchers have proposed the use of machine learning and deep learning algorithms and intrusion detection systems to detect and respond to cyber threats in real time [25]. These systems can analyze network traffic and device behavior to send out an alert and identify potential security incidents, such as unauthorized access attempts or malware infections [10,26,27]. 

The alerts generated by the current systems are typically based on rigid, manually defined, rule-based security measures. This approach, however, is unsuitable for heterogeneous and dynamic systems such as the IoMT, where both normal and malicious profiles continuously change based on the context [28]. For instance, the acceptable range for the heart rate during relaxation can differ substantially from that during physical activities such as running or walking. Given that a patient’s movement and health condition can be unpredictable, these systems may generate numerous false alerts. Therefore, a machine learning approach is more appropriate for developing an intelligent and resilient IDS. Our study aims to enhance the accuracy of such machine learning-based IDS by addressing a prominent issue that negatively affects detection accuracy—overfitting.

Machine learning- and deep learning-based solutions have emerged as promising approaches to address the cyber threats faced by the Internet of Medical Things (IoMT) [29,30]. These algorithms can analyze large amounts of data and identify patterns and anomalies that may indicate a security incident [31]. For example, deep learning models can be used to detect and respond to network intrusions in real time by analyzing network traffic and identifying unusual behavior that may indicate an attack [30]. Additionally, deep learning models can be trained to detect malware infections and prevent them from spreading by identifying malicious behavior and blocking it before it causes harm [25,32]. This is particularly important in healthcare environments, where malware infections can have serious consequences for patient safety and privacy [33]. As the healthcare industry has experienced several major ransomware attacks, special attention needs to be paid to protect the IoMT to detect such threats at the early stages [18,34]. 

The use of deep learning algorithms for anomaly-based intrusion detection systems (IDS) in the Internet of Medical Things (IoMT) has gained significant attention in recent years [35]. Anomaly-based IDS aim to detect security incidents by identifying deviations from normal behavior in the network or devices [36]. Deep learning algorithms can be used to model this normal behavior and detect anomalies in real time. In the field of IoMT security, deep learning algorithms can also be used to perform automated feature selection, which can help to reduce the risk of overfitting and improve the performance of intrusion detection systems [23,37]. Additionally, deep learning algorithms can be used to generate representations of the data that can be used as inputs for intrusion detection models, allowing these models to learn more effective representations of the data and improve their accuracy in detecting cyber threats [38]. However, embedding the feature selection functionality into deep learning needs sufficient data and attack behavior to work properly, which is not the case for early detection where the data is not fully captured yet [39,40]. Another approach is to conduct feature selection as a preprocessing step before model training [41]. Such an approach helps to remove noise and reduce the data dimensionality.

Recent studies have shown that feature selection is crucial for improving the performance of intrusion detection systems (IDS) in various IoT environments [42,43,44]. One of the most commonly used feature selection techniques is the filter approach, which selects features based on their statistical properties and relevance to the problem [45]. Filter methods for feature selection in intrusion detection systems (IDS) in IoT environments are based on the statistical properties and relevance of features to the problem [44]. Some commonly used filter methods include the chi-square test, information gain, and mutual information [46]. These methods assess the significance and relevance of each feature to the class label and rank the features based on their scores. The top-ranked features are then selected for use in the IDS. The advantage of filter methods is that they are computationally efficient and do not require training the IDS, making them suitable for large-scale IoT networks [47].

Mutual information feature selection is a commonly used filter method for improving the performance of intrusion detection systems (IDS) in various IoT environments [46,48]. This method measures the dependence between each feature and the class label and selects the features with the highest mutual information scores [44]. The main advantage of using mutual information is that it considers the nonlinear relationships between features and class labels, making it suitable for handling complex and nonlinear data patterns in IoT environments [48,49]. Furthermore, mutual information is also able to handle noisy and incomplete data, which are commonly encountered in IoT networks [48,50]. By selecting the most relevant and informative features, mutual information feature selection can effectively reduce the dimensionality of the data and improve the accuracy of the IDS. Several studies have demonstrated the effectiveness of mutual information feature selection for IDS in IoT environments and have compared its performance with other feature selection methods [45,49,51].

The relevance–redundancy trade-off is a common issue encountered in mutual information feature selection for intrusion detection systems (IDS) in IoT environments [52]. On the one hand, it is important to select features that are relevant to the class label and provide important information for the IDS. On the other hand, including redundant features can negatively affect the performance of the IDS by increasing the dimensionality of the data and introducing noise into the feature space. To address this trade-off, the mutual information feature selection method typically employs a threshold-based approach to balance the selection of relevant and nonredundant features. By using redundancy coefficients, mutual information feature selection can effectively handle the trade-off between relevance and redundancy and improve the accuracy of the IDS in IoT environments.

However, the current calculation of the redundancy coefficient is not suitable for the early detection of attacks, as the data collected have no sufficient attack patterns [48]. The redundancy–relevancy trade-off, achieved by adjusting the redundancy coefficients, works well for data with complete observations about attacks but may generate suboptimal feature sets when dealing with data lacking sufficient attack patterns. This is due to the reliance on mutual information calculations between candidate features and the common characteristics of already-selected features. Incomplete data, as encountered during the early phases of attacks, make it difficult to perceive such common characteristics, resulting in the inclusion of redundant and irrelevant features. Although some studies [48] have tried to address this issue, those solutions assumed that the redundancy score increases linearly with the size of the already-selected set. This assumption does not hold, as the amount of information a feature carries about the attack pattern varies from feature to feature, especially when dealing with early patterns where data are sparse, which makes it difficult to perceive the common characteristics of selected features. This negatively affects the ability of the mutual information feature selection (MIFS) goal function to estimate the redundancy coefficient accurately. To overcome this issue, this paper proposes an enhanced feature selection technique called Logistic Redundancy Coefficient Gradual Upweighting MIFS (LRGU-MIFS) that evaluates candidate features individually instead of comparing them with common characteristics of the already-selected features. Unlike existing feature selection techniques, LRGU calculates the redundancy score of a feature using the logistic function. It increases the redundancy value based on the logistic curve, which captures the nonlinearity of the relationship of the mutual information between features in the selected set. 

The contribution of this paper is three-fold.

1. We propose an improved redundancy–relevancy tradeoff technique for the goal function of the MIFS.
2. We integrate the improved MIFS into the training phase of the IDS model for the IoMT.
3. We conduct an experimental evaluation to measure the accuracy of our improved model and compare it with the existing solutions.

The rest of the paper is organized as follows. Section 2 discusses the related works. Section 3 describes the methodology adopted by our study. Section 4 explains the experimental evaluation, results, and analysis. The paper ends with a conclusion paragraph that highlights the contributions and suggestions for further research.

## 2. Related Works

The IoMT can be applied in several healthcare applications to improve human–robot interactions during surgical operations, for wearable respiratory and activity monitoring, and in network architecture for healthcare applications [53,54,55,56]. The study conducted by [53] developed a multi-sensor guided hand gesture recognition system for surgical robot teleoperation that employs a Long Short-Term Memory Recurrent Neural Network (LSTM-RNN) classifier for an increased recognition rate and faster inference speed. Additionally, a wearable respiratory and activity monitoring (WRAM) system was proposed to understand breathing patterns during daily activities [54]. The system uses a multimodal fusion architecture and a hybrid hierarchical classification algorithm to distinguish complex activities and calculate the respiratory and exercise parameters. In surgical applications, a human activity-aware adaptive shared control solution was introduced to achieve a smooth transition between hands-on control and teleoperation, improving surgical workflow and safety [55]. Lastly, a Cybertwin-based multimodal network [56] was proposed for monitoring electrocardiogram (ECG) patterns during daily activities, employing a deep convolutional neural network for enhanced identification accuracy. These advancements in human–robot interactions, wearable monitoring systems, and network architecture contribute to the development of more effective, safer, and personalized healthcare solutions.

The IoMT architecture is a combination of IoT technology in the medical domain following a three-tier structure: a perception layer, network layer, and application layer. The perception layer focuses on data acquisition and data access, using various equipment and technologies to collect and transmit data from people and things. The network layer comprises the network transmission and service layers, which enable data transmission and the integration of heterogeneous networks, data formats, and data warehouses. Lastly, the application layer manages medical information and medical decision-making applications, covering various aspects of patient care and analysis. Cybersecurity challenges in the IoMT architecture involve securing the data acquisition process, protecting sensitive information, and ensuring robust access controls during transmission and integration.

At each layer of the IoMT architecture, unique cybersecurity challenges emerge. The perception layer requires safeguarding the data acquisition and transmission processes from unauthorized access or tampering. In the network layer, maintaining data integrity and confidentiality during transmission, securing the integration of heterogeneous networks, and implementing robust access controls to protect open interfaces are essential. Lastly, the application layer must prioritize the protection of patient data privacy and implement strong access control mechanisms to prevent unauthorized access to critical medical systems and decision-making applications. Addressing these cybersecurity challenges is vital for ensuring the reliability, privacy, and trustworthiness of IoMT systems.

The existing alert systems used in the IoMT primarily rely on rule-based mechanisms, which are not ideal for detecting evasive behavior where attack patterns continuously change. Rule-based systems have limitations when dealing with the dynamic nature of the IoMT, as they use static rules to protect constantly evolving environments. This dynamicity quickly invalidates fixed rules, rendering them ineffective in providing adequate security.

An alternative to rule-based systems is the implementation of more intelligent, dynamic, and adaptive intrusion detection systems. These systems leverage machine learning and artificial intelligence techniques to analyze network traffic patterns and user behavior, enabling them to detect anomalies and potential threats. Unlike static rule-based systems, these intelligent systems can adapt to the changing nature of IoMT environments by continuously learning from new data and updating their models. Some examples of such intelligent intrusion detection systems include those based on deep learning, such as Long Short-Term Memory (LSTM) and convolutional neural networks (CNN), which can automatically learn and identify complex patterns in data. Another approach is the use of unsupervised learning algorithms, such as clustering and anomaly detection, which can detect unusual behavior without prior knowledge of specific attack patterns.

The field of intrusion detection systems (IDS) for the Internet of Medical Things (IoMT) has gained significant attention in recent years due to the increasing use of IoMT devices in healthcare systems. The development of effective IDS is critical to ensure the security and privacy of sensitive medical data. Many studies have focused on developing IDS for traditional networks, but there is a lack of research on IDS for the IoMT [15]. In this section, we will review the related works on IDS for the IoMT, with a focus on feature selection methods. We will also discuss the limitations of the current IDS and identify gaps in the literature that need to be addressed in future research.

Feature selection techniques play an important role in improving the performance of intrusion detection systems (IDS) in the Internet of Medical Things (IoMT) [25,35]. There are various feature selection algorithms that have been used in the literature to reduce the dimensionality of the input features while preserving their relevant information [36]. Filter-based methods, such as chi-square and information gain, evaluate each feature individually to determine its contribution to the target variable [30]. Wrapper-based methods, such as recursive feature elimination (RFE), use a machine learning algorithm to iteratively select and remove features based on their impact on the performance of the model [30]. This approach measures the contribution of each feature to the model’s prediction. Therefore, wrapper feature selection techniques can be combined with other machine learning algorithms to create an effective IDS for the IoMT. Embedded methods, such as Lasso and Random Forest, perform feature selection as a part of the training process [37]. 

Information theory-based feature selection is a type of filter-based method that uses information theory concepts, such as entropy and mutual information, to assess the relevance of features [18,37]. This approach evaluates the relationship between the features and the target variable and selects only the most informative features [18,38]. Information theory-based feature selection algorithms, such as Mutual Information-based Feature Selection (MIFS) and Maximum Relevance–Minimum Redundancy (MRMR), have been applied in various domains, including intrusion detection in the IoMT [38,39]. These methods have been found to be effective in improving the performance and efficiency of IDS in the IoMT by reducing the dimensionality of the input data while preserving the relevant information [40]. Information theory-based feature selection techniques are well suited for IDS in the IoMT, as they can effectively handle the high-dimensional and complex data commonly encountered in this domain [33].

One of the main issues with Mutual Information-based Feature Selection (MIFS) and other information theory-based feature selection techniques is the lack of sufficient data [32]. In order to accurately evaluate the mutual information between the features and the target variable, large amounts of data are required. When the data are limited, the estimate of the mutual information can be imprecise, leading to suboptimal feature selection results [41]. 

There have been several proposed solutions to the issue of data insufficiency in Mutual Information-based Feature Selection (MIFS) and other information theory-based feature selection methods. One approach is to use data augmentation techniques, such as oversampling or synthetic data generation, to increase the amount of available data [42]. Another approach is to use transfer learning, where the knowledge learned from one domain is transferred to another domain with limited data [43]. This can help to improve the performance of the feature selection algorithm by leveraging the information from related domains with more data.

While augmentation-based solutions, such as oversampling or synthetic data generation, can help to increase the amount of available data and improve the performance of information theory-based feature selection methods, there are also potential drawbacks to consider. One potential issue with augmentation-based solutions is the risk of introducing bias or noise into the data [44]. Oversampling, for example, can lead to the overrepresentation of certain classes or instances in the data, which can bias the machine learning algorithm towards these classes or instances. 

Synthetic data generation, on the other hand, may not accurately represent the underlying data distribution and may introduce noise into the data, which can negatively impact the performance of the feature selection algorithm [45]. Another potential issue with augmentation-based solutions is the computational cost. Oversampling or synthetic data generation can be computationally expensive, especially for large datasets, and may require significant computational resources [46]. This can increase the overall time and cost of the feature selection process and may limit the scalability of the intrusion detection system. Moreover, augmentation-based solutions may not always be feasible or practical in real-world settings. For example, in the IoMT, it may not be possible to obtain or generate additional data due to privacy or ethical concerns. In these cases, augmentation-based solutions may not be a viable option, and alternative solutions may need to be considered.

The redundancy–relevancy trade-off for information-theoretic feature selection is another approach that focuses on improving the goal function of the feature selection technique [32,47]. The technique improves feature selection based on a redundancy coefficient measure that quantifies the extent to which each feature contributes to the joint mutual information with other features [47]. The approach involves gradually upweighting the contribution of each feature based on its redundancy coefficient to overcome the limitations of the traditional information theory-based feature selection methods. The approach is based on the idea that the degree of redundancy between a candidate feature and the already-selected features can be quantified individually in isolation from the other features. This gives the feature selection the flexibility to accurately calculate the significance of the candidate feature. However, this approach assumes that redundancy increases linearly with the size of the selected set. This assumption is not realistic, as the correlation between features is not linear, especially when data are insufficient, as is the case for early attack detection.

## 3. Materials and Methodology

Our study primarily aims to enhance the accuracy of DS by addressing the issue of redundant features that could potentially trigger overfitting, subsequently reducing the accuracy of machine learning-based IDS systems. The current security measures predominantly rely on rule-based methods, where fixed, predetermined firewall policies are enforced to safeguard the IoMT system. However, the dynamism, heterogeneity, and complexity of the IoMT environment complicate the task of discerning hidden relationships between attributes. This complexity becomes particularly challenging in the face of evasive attacks, where threat actors attempt to conceal their activities by mimicking regular operations, thereby bypassing standard rule-based security measures. Consequently, there is a need for more sophisticated IDS solutions. These systems should be capable of identifying evasive attack patterns and features while maintaining minimal system complexity and avoiding overfitting that could adversely affect IDS accuracy.

**Figure 1:**
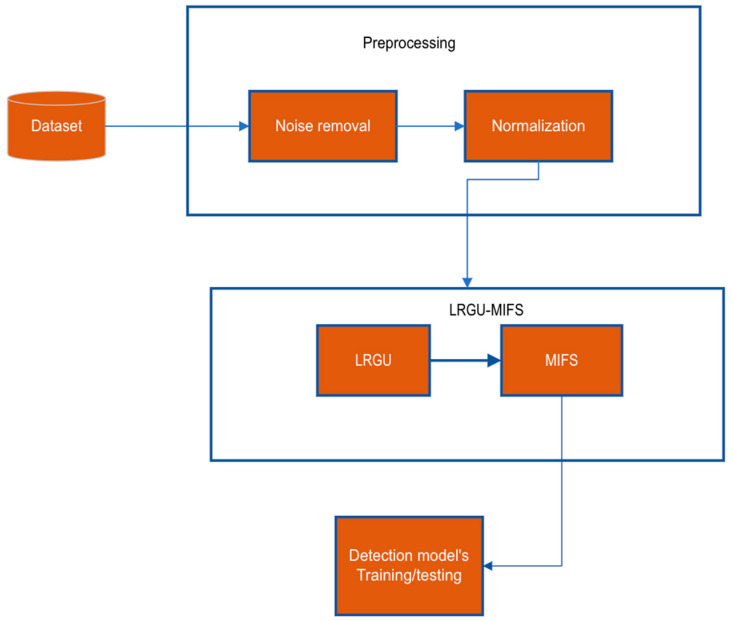
Methodology diagram.

The methodology section of this paper outlines the steps and techniques used to develop a Logistic Redundancy Coefficient Gradual Upweighting (LRGU)-based feature selection technique that can be used in IDS for the Internet of Medical Things (IoMT). The LRGU is integrated into mutual information feature selection (MIFS). The goal of this study is to design an effective and efficient LRGU-MIFS technique to select the relevant features and improve the IDS, which, in turn, protects devices and networks in the IoMT against cyber threats. This methodology section will provide a detailed explanation of the data preprocessing, model design, and the evaluation method used to evaluate the performance of the proposed IDS. The focus is on explaining the technical aspects of the LRGU-MIFS, including the data representation, feature selection, and technique architecture, to provide a clear understanding of the methodology used in this study. As shown in Figure 1, our methodology starts by preprocessing the data, which involves noise removal that filters out the noise data, then normalization that puts all data in the range between 0 and 1. This step is necessary to prepare the data for the next stage, where it will be used as input for the LRGU-MIFS technique. The LRGU-MIFS selects the relevant and nonredundant features with the aid of the LRGU redundancy coefficient, which helps to estimate the redundancy score of the candidate features more accurately than the related works. Once the features are selected, they will be used to train the machine learning classifier; using which, the relevancy of these features can be measured and compared with those selected by the existing methods.

### 3.1. Data Preprocessing

Preparatory data processing, such as removing the noise, missing data imputation, and normalization, has been performed to get the data ready for modeling. During the preprocessing stage, we took meticulous steps to ensure the integrity of the data range, sequence of values, and collection intervals. Firstly, to maintain data range loyalty, we applied the normalization technique that preserved the original range of data while transforming it into a more suitable form for our machine learning algorithms. For sequence preservation, we did not randomize or reorder the dataset during preprocessing, ensuring that the temporal order of the datapoints was maintained, which is particularly critical for time series data such as ours. Finally, to maintain the same collection intervals, we did not perform any resampling operations that could change the original data frequency. Instead, we worked within the existing time intervals provided by the original data collection process. These careful preprocessing measures ensured that our data’s essential characteristics were preserved, thus upholding the reliability of our subsequent analysis and results.

Noise removal is an important step in data preprocessing to ensure that the data are clean and free of irrelevant or misleading information. Noise in the data can be introduced in a variety of ways, such as measurement errors, missing values, or outliers. Such noises can negatively impact the performance of the IDS model, causing it to generate incorrect or unreliable decisions. A statistical mean and standard deviation-based filter was used to identify and remove the outliers in each attribute of the dataset.

Normalization scales all attribute values to a range between 0 and 1 to prevent the machine learning algorithm from overemphasizing attributes with larger ranges during the training phase. This helps to eliminate the influence of large values on the training process, which can cause the algorithm to favor certain features over others. Additionally, normalization helps to improve the convergence of the algorithm and to reduce the risk of overfitting. It also helps to ensure that the model is not biased towards any particular feature and provides a more accurate representation of the relationships between the features in the dataset. Equation (1) was used for data normalization.

(1) $$X_{norm}=\left(X-X_{min}\right)\;/\;\left(X_{max}-X_{min}\right)$$

where *X* is the original value, $X_{min}$ is the minimum value in the dataset, $X_{max}$ is the maximum value in the dataset, and $X_{\mathrm{norm}}$ is the normalized value. This formula scales the value of X to the range between 0 and 1 by subtracting the minimum value and dividing it by the range of the data $\left(X_{max}-X_{min}\right)$. This normalizes the data and makes the scales of the features consistent across the entire dataset.

### 3.2. An Enhanced Mutual Information Feature Selection Technique

As pointed out in the related works section above, MIFS is a popular technique for feature selection that can effectively select relevant features regardless of data distribution, which makes it suitable for early detection scenarios where data have no sufficient attack patterns. For two discrete variables, mutual information (*MI*) is a measure of how much information the variables share about each other. The calculation of *MI* is given by Equation (2).

(2) $$MI\left(X;Y\right)=H\left(X\right)-H(X|Y)=\underset{y\epsilon Y}{\sum }\underset{x\epsilon X}{\sum }p\left(x,y\right)\log\frac{p\left(x,y\right)}{p\left(x\right)p\left(y\right)}$$

where *H*(*X*) is the entropy of *X*, *H*(*X*|*Y*) is the conditional entropy of *X* given *Y*, *p*(*x*) and *p*(*y*) are the marginal distributions of *x* and *y*, and *p*(*x*, *y*) is the joint distribution of *x* and *y*. The entropy *H*(*X*) is calculated using Equation (3).

(3) $$H\left(X\right)=-\underset{x_{i}\in X}{\sum }p\left(x_{i}\right)\log\left(p\left(x_{i}\right)\right)$$

The conditional entropy *H*(*X*|*Y*) is calculated using Equation (4).

(4) $$H\left(X|Y\right)=-\sum_{y_{j}\in Y}p\left(y_{j}\right)\sum_{x_{i}\in X}p\left(x_{i}|y_{j}\right)\log\left(p\left(x_{i}|y_{j}\right)\right)$$

The general formula for the linear combinations of Shannon information terms is represented by Equation (5) (Brown et al. (2012) and Li et al. (2017)).

(5) $$J\left(X_{k}\right)=I\left(X_{k};Y\right)-\beta \underset{X_{j}\epsilon S}{\sum }I\left(X_{j};X_{k}\right)+\gamma \underset{X_{j}\epsilon S}{\sum }I\left(X_{j};X_{k}|Y\right)$$

This equation is composed of a relevancy term and a redundancy term, represented by Expressions (6) and (7), respectively. The relevancy term is represented by Expression (6), while the redundancy term is represented by the sum of marginal redundancy shown in Expression (8) and conditional redundancy shown in Expression (9), both being weighed by parameters β and γ with values between 0 and 1.

(6) $$I\left(X_{k};Y\right)$$

(7) $$\beta \underset{X_{j}\epsilon S}{\sum }I\left(X_{j};X_{k}\right)+\gamma \underset{X_{j}\epsilon S}{\sum }I\left(X_{j};X_{k}|Y\right)$$

(8) $$\beta \underset{X_{j}\epsilon S}{\sum }I\left(X_{j};X_{k}\right)$$

(9) $$\gamma \underset{X_{j}\epsilon S}{\sum }I\left(X_{j};X_{k}|Y\right)$$

The mutual information between the candidate feature $X_{k}$ and the class label *Y* is expressed by $I\left(X_{k};Y\right)$, while the conditional mutual information between $X_{k}$ and other features $X_{j}$ in the selected set *S* given the class label *Y* is expressed by $I\left(X_{j};X_{k}│Y\right)$.

### 3.3. Logistic Redundancy Coefficient Gradual Upweighting

This section describes our proposed Logistic Redundancy Coefficient Upweighting (LRGU) technique that is used for the redundancy estimation of the features. The magnitude of coefficient *β* establishes the extent of trust in the redundancy term. Unlike the existing MIFS that computes *β* using Equation (10), we propose a Logistic Redundancy Gradual Upweighting (LRGU) that computes the redundancy using Equation (11). Instead of updating the value of *β* linearly, the LRGU gradually increases the weights every time a new feature is added to the selected set. Meanwhile, our approach does not discard the potential common characteristics between the features in the selected set. Concretely, the value of *β* starts from 0.5 and increases gradually to 1, thanks to the logistic function in Equation (11) as the redundancy coefficient. This means that the value of *β* starts as low as 0.5 at the beginning of the selection process, reflecting the small size of the initially selected set *S*. The value of *β* then gradually increases as the size of set *S* grows.

(10) $$\beta =\frac{1}{\left|S\right|}\;\;\;$$

(11) $$\beta =\frac{1}{1+e^{-\left(\frac{\left|S\right|}{\left|F\right|}\right)}}\;\;$$

The number of features in the selected set and the original set are represented by |*S*| and |*F*|, respectively. 

The proposed LRGU increases the redundancy value according to the logistic curve, which ranges between 0 and 1. When the size of the feature set is small, the redundancy value approaches its lower bound of 0. Conversely, when *x* is the size of the selected set increase, the redundancy approaches its upper bound of 1. By substituting *β* in Equation (7) with the LRGU, the improved MIFS will calculate the feature significance according to Equation (12), as follows.

(12) $$I\left(X,Y\right)=\frac{1}{1+e^{-\left(\frac{\left|S\right|}{\left|F\right|}\right)}}\;\;\;\sum_{X_{j}\epsilon S}I\left(X_{j};X_{k}\right)+\gamma \sum_{X_{j}\epsilon S}I\left(X_{j};X_{k}|Y\right)\;$$

This calculation is different from the approach used in [48], which calculated the redundancy score using Expression (13), where the redundancy increase linearly follows the increase of the selected feature set size.

(13) $$\frac{\left|S\right|}{\left|F\right|}\;$$

### 3.4. LRGU-Based Mutual Information Feature Selection

Equation (12) shows how the LRGU is incorporated into the MIFS. It ensures that, during each iteration, the feature with the highest mutual information with the class label, given the already selected features, is added to set *S*. The pseudocode in Algorithm 1 shows how the proposed LRGU-MIFS works. The technique starts with calculating the mutual information value for each feature in the original set *F*. The feature with the highest mutual information value is then selected and stored in *S*. The subsequent features are added into the selected set based on Equation (14). After that, the features in *S* are ranked from highest to lowest according to their *J (*$x_{k}$*)* value, and the top *n* features are retained, while the others are discarded. The value of *n* is determined based on the desired number of features.

(14) $$LRGU\_MIFS\left(x_{k}\right)=MI\left(x_{k},y\right)-\frac{1}{1+e^{-\left(\frac{\left|S\right|}{\left|F\right|}\right)}}\;\left(\underset{s_{j}\epsilon S}{\sum }I\left(x_{k},x_{j}\right)\right)\;\;\;\;\;\;\;\;\;\;\;$$

**Algorithm 1.** Pseudocode for the LRGU-MIFS.**Input:** $F=\left\{f_{1},\;f_{2},\;\ldots ,f_{a}\right\}$ entire features set; C  class label, n required features. **Output:** $S=\left\{s_{1},\;s_{2},\ldots .,s_{b}\right\}$ the selected features set.
**1:** G←∅; S←∅//start with empty lists **2:** for each feature $f_{i}\in F$: //calculate the MI score **3:**      $g_{i}=MI\left(f_{i};C\right)$ **4:**      $G\leftarrow G\cup g_{i}$ **5:** $g_{k}\leftarrow \max\left(G,MI\right)$     // pick the feature with the highest MI score **6:** $S\leftarrow g_{k}$; $G\leftarrow G\backslash \left\{g_{k}\right\}$// add the feature with the highest MI score into the selected list and remove it from the original list **7:** for $\forall \;\left(g_{j},s_{m}\right)$ with $g_{j}\in G$ and $s_{m}\in S$// calculate the LRGU-MIFS **8:**      compute $MI(C;s_{m}|g_{j})$ **9:**      $s_{d}=\underset{v_{j}\in V}{\mathrm{argmax}}\left[\sum_{s_{m}\in S}LRGU\_MIFS(C;g_{j}|s_{m})\right]:$ **10:**      $G\leftarrow G\backslash \left\{s_{d}\right\}$//to remove the selected feature from original list **11:**      $S\leftarrow S\cup \{s_{d}\}$// add the selected feature to the selected list **12:** Repeat 8–11 while size(S)≤n


### 3.5. The Dataset

In this paper, we used the WUSTL-EHMS-2020 dataset that contains data from network flow metrics and patients’ biometrics. The dataset was created based on a real-time Enhanced Healthcare Monitoring System (EHMS) testbed. The testbed is composed of four components: medical sensors, a gateway, a network, and a control and visualization component. The data flow in the system begins at the sensors attached to the patient’s body and moves to the gateway, which then sends the data to the server for visualization through a switch and router. 

The EHMS testbed collects both the network flow metrics and patients’ biometrics. It is comprised of six key components: a multi-sensor board, a gateway, a server, an intrusion detection system (IDS), an attacker, and a network. The PM4100 Six Pe Multi-Sensor Board, a product from Medical Expo, is equipped with four sensors to monitor a patient’s vital biometric data, including heart electricity (ECG), blood oxygen saturation (SpO_2_), body temperature, and blood pressure. These data are transmitted via a USB port to a Windows-based laptop, or gateway, which presents the data on a graphical user interface (GUI) and sends it to the server for further processing. The server is an Ubuntu-based laptop that collects and analyzes the data to aid in medical decision-making. The network is comprised of an Ethernet switch connecting the server, IDS, and attacker computer, with a router assigning dynamic IP addresses. The IDS computer uses Argus network flow monitoring software to collect network flow metrics and biometric data and makes decisions regarding traffic packets. Lastly, the attacker is a Kali Linux-based computer used to simulate potential attacks on the system, such as spoofing and altering patient data during transmission, to mimic potential risks in healthcare monitoring systems.

The environment is structured to replicate a realistic IoMT setting in which medical sensors are attached to a patient’s body to collect vital biometric data, such as heart rate, blood pressure, and oxygen levels. These sensors continuously monitor the patient’s health status and transmit the collected data to a gateway device.

The gateway serves as an intermediary between the medical sensors and the network infrastructure, receiving data from the sensors, processing it, and forwarding it to the server via a switch and router. This ensures seamless data transfer and effective communication between the different components of the system. The network infrastructure comprises various devices, such as switches, routers, and firewalls, that facilitate data transmission from the gateway to the server.

In the control and visualization component, the server receives the data and presents it in a user-friendly interface, allowing healthcare professionals to monitor and analyze the patient’s health status. This real-time visualization aids medical staff in making informed decisions regarding the patient’s treatment and care.

The WUSTL EHMS 2020 Dataset collects a comprehensive range of biometric data using a system that comprises six building blocks: a multi-sensor board, a gateway, a server, an IDS, an attacker, and a network [7]. The PM4100 Six Pe Multi-Sensor Board collects a patient’s biometric data, including ECG, SpO_2_, body temperature, and blood pressure, to monitor a patient’s health. The data are gathered using an electrocardiogram (ECG or EKG) sensor, which measures the heart’s electrical activity through three electrode pads. A blood oxygen saturation (SpO_2_) sensor measures the oxygen levels in the blood and heart rate, where values between 95 and 100 percent are considered normal. Additionally, temperature sensors record the body temperature, and blood pressure sensors employ a stepwise gassing method to measure the systolic and diastolic arterial pressure. The critical parameters collected include the heart rate (HR), respiration rate (RR), the electrically neutral area between ventricular depolarization and repolarization (ST), systolic (SYS) and diastolic (DIA) blood pressure, blood oxygen (SPO_2_), pulse rate (PR), and body temperature (TEMP). This dataset provides a holistic view of the patient’s health, enabling healthcare professionals to make informed decisions regarding their care.

The data are then sent to a Windows-based laptop serving as the gateway, which presents the information on a GUI and transmits it to an Ubuntu-based server for further analysis. A regular Ethernet switch connects the server, IDS, and attacker computer in a single network, with a router assigning dynamic IP addresses. The IDS computer runs Argus network flow monitoring software to analyze the network flow metrics and biometric data, making real-time decisions to detect any abnormalities. Finally, a Kali Linux-based computer acts as an attacker, simulating cyber-attacks on the system by spoofing and altering a patient’s biometric data during transmission over the network using the Python script and the Scapy library.

The dataset was collected using the Audit Record Generation and Utilization System (ARGUS) tool [2]. The dataset had 44 features in total, with 35 network flow metrics, 8 patient biometric features, and 1 feature serving as the label. 

In this dataset, the attacks against the EHMS were initiated using tools in Kali Linux. These attacks tried to spoof and alter patient biometric data during transmission across the network. The attacks were executed using the Python script and the Scapy library, which provides capabilities such as live connection sniffing, packet spoofing, and real-time packet alteration. This library has extensive support for various protocols and offers numerous tools for analyzing network and host security.

### 3.6. Experimental Environment and Evaluation Metrics

To implement the proposed LRGU-MIFS and evaluate its performance, the development and experimental evaluation were conducted using several tools and software, including Python, Skfeature, TensorFlow, Keras, Scikit Learn, and NumPy. Moreover, the preparation of data samples, implementation of algorithms, and the analysis of the results were carried out on a machine with an Intel(R) Core (TM) i7-4790 CPU @ 3.60 GHZ and 16 GB RAM.

In order to assess the efficacy of the proposed LRGU-MIFS, this paper used accuracy as the evaluation metric. The approximation error of the IDS model was also assessed by its false positive and false negative rates, which are widely used measures in the existing research. The detection accuracy was calculated using Equation (15).

(15) $$ACC=\frac{TP+TN}{TP+TN+FP+FN}$$

where *TP*, *TN*, *FP*, and *FN* denote true positive, true negative, false positive, and false negative, respectively.

## 4. Results and Discussion

In this section, the results of the proposed LRGU-MIFS technique are discussed, along with comparisons with the related works. The experimental evaluation was conducted using several Python-based packages, including SkLearn, Pandas, NumPy, and SkFeature. To measure the performance of our technique, several machine learning classifiers were used, namely Support Vector Machines (SVM), Logistic Regression (LR), Random Forest (RF), Decision Tree (DT), and Long Short-Term Memory (LSTM), using the features selected by LRGU-MIFS. The following steps were involved in training the models for intrusion detection in the IoMT using a set of features selected by LRGU-MIFS. The first step was to project the selected features to the dataset, which contained both normal and anomalous instances of network traffic in the IoMT environment. The next step was to preprocess the data. This included normalizing the data, handling missing values, and transforming the data into a format suitable for training the model. Once the data were preprocessed, they were split into training and validation sets. The training set was used to train the five classifiers, while the validation set was used to evaluate its performance. Then, the classifier parameters were defined. This included choosing the number of layers, the number of neurons in each layer, the activation function, and the optimizer. After defining the model architecture, it was trained using the training set. During training, the model was updated based on the prediction error, which was calculated using the loss function. After the training, the model’s performance was evaluated using the validation set. This included calculating the accuracy, precision, recall, and other relevant metrics to assess the model’s performance.

The experiments were conducted using several feature sets with a different number of features, i.e., 5, 10, 15, 20, 25, 30, 35, 40, and 45 features. Several machine learning classifiers were used, namely Support Vector Machines (SVM), Logistic Regression (LR), Random Forest (RF), Decision Tree (DT), and Long Short-Term Memory (LSTM). These classifiers were chosen since they are suitable for binary classification and cover various scenarios. The dataset was divided into a training set and a testing set using a 10-fold cross-validation approach. The testing set then was used to determine the classification accuracy of those classifiers.

Table 1 shows the accuracy results for each classifier in each feature set. It can be observed that the accuracy increased when the size of the feature set increased from 5 to 10 features. Except for the DT that started with an accuracy level of 0.859, all classifiers achieved an accuracy of around 0.88 using a feature set size of 5. When the size of the features increased to 10, the accuracy of all the classifiers increased to around 0.93 (SVM, LR, and LSTM); 0.943 (DT); and 0.925 (RF). When the number of features increased to 45, the accuracy fluctuated between 0.933 and 0.934 (SVM and LSTM), 0.922 and 0.925 (RF), and 0.939 and 0.949 (DT). Moreover, the accuracy of LR did not change when the number of features increased. 

The results in Table 1 show that improvement of the detection accuracy was achieved when the models were trained with the top 10 features. When more features were added, the improvement was not significant. This indicates that the proposed LRGU-MIFS was able to identify a compact set containing the most relevant features. This can be attributed to the LRGU-MIFS’s ability to capture the nonlinear relationship of mutual information between selected features, enabling it to accurately estimate feature significance while striking an optimal balance between relevancy and redundancy. In addition, it could be observed that, when adding more (less significant), the accuracy did not improve. This is because of the overfitting that these features cause to the model, as they only increase the data dimensionality while adding little information to the relationship between input data and the label. 

However, it is worth noting that the rate of improvement diminishes when additional features are added beyond this point, indicating that the effect of redundancy becomes more influential. This implies that there may be a limit to the benefits gained by increasing the number of features, as the redundancy starts to counteract the advantages.

**Figure 2:**
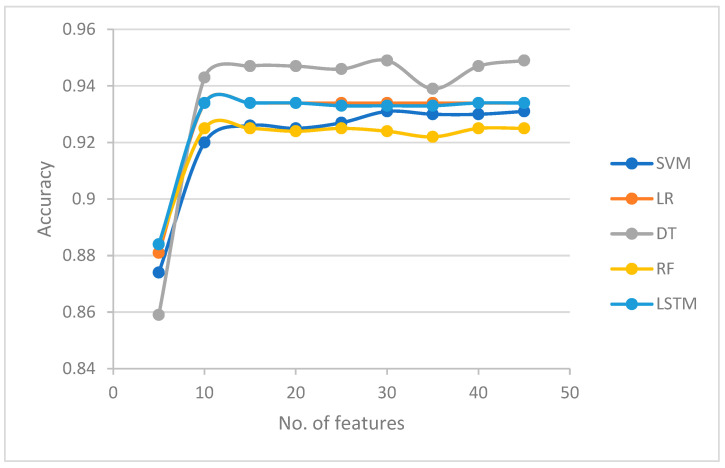
The no. of features in the accuracy ratio.

Figure 2 investigates the relationship between the feature set size and classification accuracy in machine learning classifiers, with a particular focus on the nonlinear behavior of accuracy as the size of the feature set increases. The results indicate that accuracy does not increase linearly as the size of the selected set increases by a fixed number of features (in this case, five features). When training all the classifiers (except LR, where the accuracy does not change after 10 features) when the number of features not exceeding 25, the effect of overfitting dominates and overrides the information gained from the new features. These findings are visually represented in Figure 2, which demonstrates a rapid rise in accuracy at the beginning when the number of features increases from 5 to 10, followed by a less-steep rise. This indicates that redundancy becomes more influential when adding more features, supporting our assumption that the redundancy coefficient needs to be increased as the number of features increases.

Furthermore, the shape of the curve in Figure 2 resembles that of a logistic function, suggesting that the effect of redundancy follows a logistic function regime. This supports our assumption about the nonlinearity of redundancy and the use of the logistic function for redundancy calculations. These results provide important insights into the optimal selection of feature sets for machine learning classifiers, highlighting the need for the careful consideration of redundancy and its nonlinear effects when choosing feature sets.

**Figure 3:**
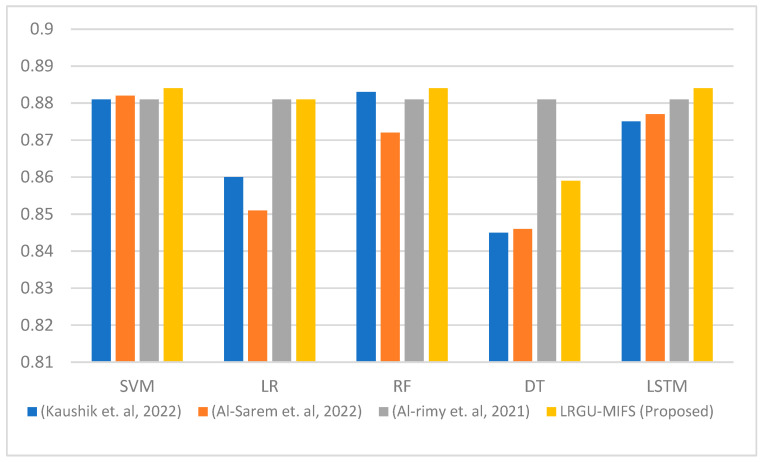
Comparisons between the accuracy of the proposed LRGU-MIFS and related features selection techniques (Al-Sarem et al. [44], Al-rimy et al. [48], Kaushik et al. [57]) using a set of five selected features.

**Figure 4:**
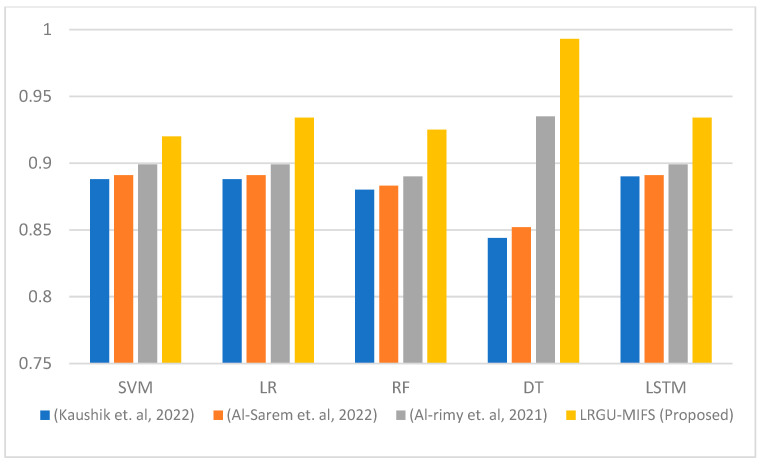
Comparisons between the accuracy of the proposed LRGU-MIFS and related features selection techniques (Al-Sarem et al. [44], Al-rimy et al. [48], Kaushik et al. [57]) using a set of 10 selected features.

**Figure 5:**
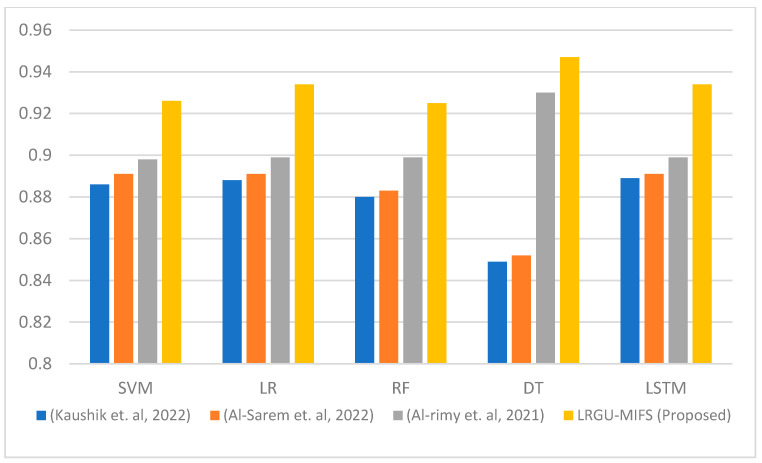
Comparisons between the accuracy of the proposed LRGU-MIFS and related features selection techniques (Al-Sarem et al. [44], Al-rimy et al. [48], Kaushik et al. [57]) using a set of 15 selected features.

**Figure 6:**
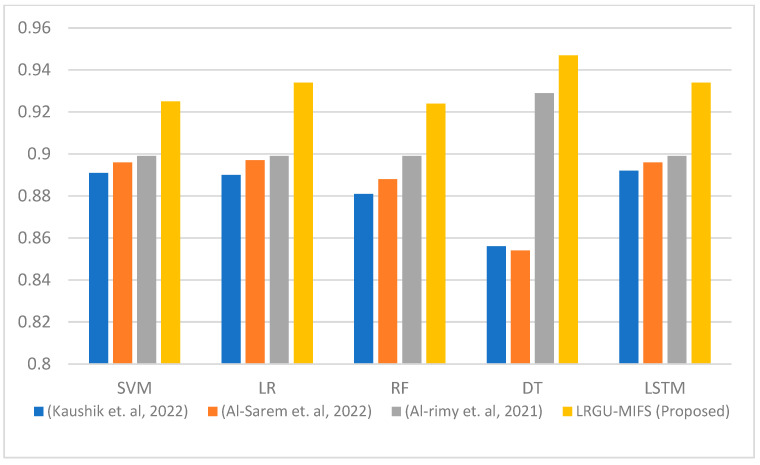
Comparisons between the accuracy of the proposed LRGU-MIFS and related features selection techniques (Al-Sarem et al. [44], Al-rimy et al. [48], Kaushik et al. [57]) using a set of 20 selected features.

**Figure 7:**
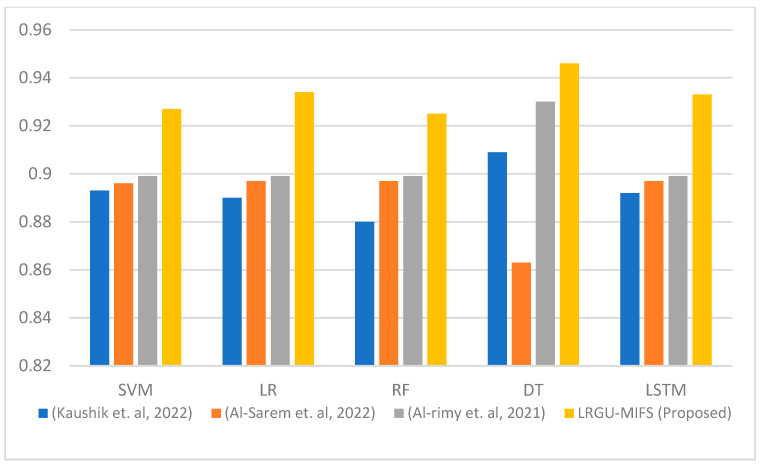
Comparisons between the accuracy of the proposed LRGU-MIFS and related features selection techniques (Al-Sarem et al. [44], Al-rimy et al. [48], Kaushik et al. [57]) using a set of 25 selected features.

**Figure 8:**
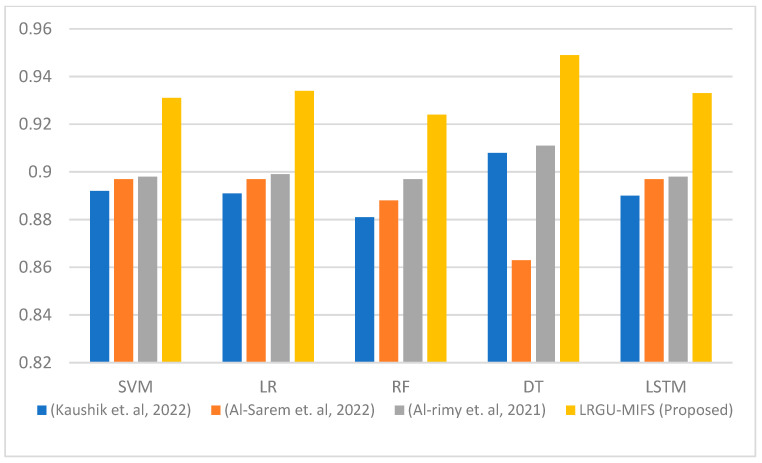
Comparisons between the accuracy of the proposed LRGU-MIFS and related features selection techniques (Al-Sarem et al. [44], Al-rimy et al. [48], Kaushik et al. [57]) using a set of 30 selected features.

Figure 3, Figure 4, Figure 5, Figure 6, Figure 7 and Figure 8 show comparisons between the accuracy achieved by the proposed LRGU-MIFS and the techniques proposed by [44,48,57]. Moreover, they show accuracy comparisons between the LRGU-MIFS and the related studies using feature sets with sizes 5, 10, 15, 20, 25, and 30, respectively. The x-axis represents the feature selection techniques, and the y-axis represents the accuracy of those techniques. The same machine learning classifiers used to evaluate the accuracy of the proposed technique were also used for comparisons with the related studies. The comparisons revealed that the proposed LRGU-MIFS achieved accuracy higher than the related technique. This advantage becomes clear when the number of features increases to 10 and above. It could also be observed that MIFS outperformed the proposed LRGU-MIFS with the DT (when trained using five features), and the accuracy of MIFS was 0.889, while the accuracy of LRGU-MIFS was 0.859. In all other instances, however, the proposed LRGU-MIFS outperformed the other techniques.

The comparison results showed that the proposed LRGU-MIFS technique outperformed the techniques used by the related works. It could be observed that the LRGU-MIFS achieved accuracy higher than the other techniques in all instances, except when training the DT with a set of five features. This is attributed to the ability of the LRGU method embedded in the goal function of the MIFS to calculate the redundancy coefficient more accurately. That is, the LRGU can capture the nonlinear relationship between the number of features in the already selected set and the redundancy score. Therefore, the goal function can estimate the feature significance with higher accuracy than the related methods that assume a linear relationship between the size of the feature set and the redundancy.

**Figure 9:**
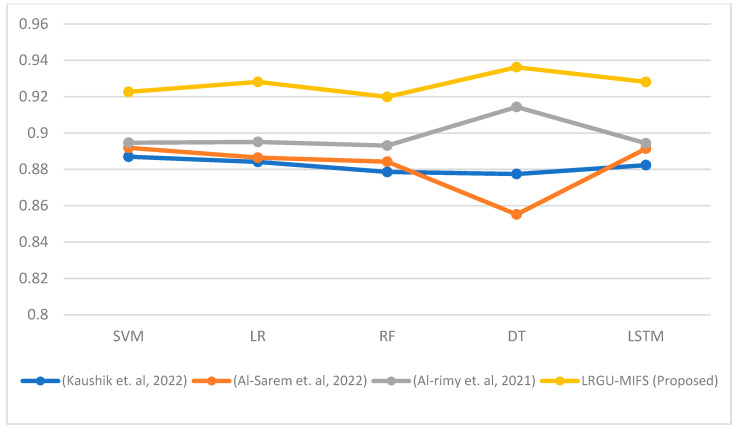
Comparisons of the average accuracy between the proposed LRGU-MIFS and related works (Al-Sarem et al. [44], Al-rimy et al. [48], Kaushik et al. [57]) compared to several machine learning classifiers.

Figure 9 displays the average accuracy across all the feature sets (ranging from 5 to 45) for various machine learning classifiers, including Support Vector Machines (SVM), Logistic Regression (LR), Random Forest (RF), Decision Trees (DT), and Long Short-Term Memory (LSTM) networks. On average, LRGU-MIFS achieved a higher accuracy than the other methods, including RCGU-MIFS [48], which employs linear redundancy upweighting. This indicates that LRGU-MIFS is classifier-independent and can be used with a variety of machine learning classifiers. It can be observed that the accuracy of the features selected by the proposed LRGU-MIFS consistently maintains a higher level than that of the other methods. This superior performance can be attributed to the nonlinear redundancy coefficient calculation utilized in LRGU-MIFS, which allows the algorithm to better account for the complex relationships between features. This enhanced capability ultimately leads to more accurate feature selection and an improved overall performance. It is worth noting that the highest accuracy average was obtained by the DT classifier for the features selected by the proposed LRGU-MIFS and RCGU-MIFS [48], as opposed to the other techniques [44,57] that did not follow the redundancy gradual upweighting approach. This can be attributed to the low redundancy of the selected features, which makes the DT able to identify the attack patterns more clearly based on those features.

## 5. Conclusions

This paper proposes an improved redundancy estimation mechanism based on Logistic Redundancy Coefficient Gradual Upweighting for feature selection in IDS for the IoMT. The technique evaluates candidate features individually to overcome the challenge of perceiving common characteristics in cases of insufficient attack patterns. This technique addresses the high data dimensionality challenge that causes overfitting and degrades the detection accuracy of the existing IDS. The experimental evaluation showed that the proposed LRGU was able to identify a compact set of significant features that outperformed those selected by the existing techniques. The LRGU outperformed the existing solutions, indicating its efficacy for a better performance. The proposed LRGU-MIFS can help to develop more accurate IDS for the IoMT and address the security concerns posed by cyber-attacks. Future works and research directions could include exploring different nonlinear redundancy estimation mechanisms to enhance the feature selection techniques for IDS in the IoMT. Evaluating the performance of the proposed LRGU-MIFS in other domains with high data dimensionality challenges, such as image processing or bioinformatics, could broaden its applicability. Investigating its performance against a wider range of attack patterns is essential for ensuring real-world robustness. Combining LRGU-MIFS with other machine learning techniques, such as ensemble learning or active learning, could further improve IDS performance. Developing a real-time IDS system for the IoMT that incorporates the proposed LRGU-MIFS would allow for evaluating its performance in detecting cyber-attacks with minimal latency and computational overhead. Additionally, examining the potential trade-offs between feature selection and preserving the privacy of sensitive patient data in the IoMT is crucial. Finally, assessing the scalability of LRGU-MIFS and its ability to handle large-scale datasets effectively will be necessary as the volume of data generated by IoMT devices continues to grow.

## Figures and Tables

**Table 1:** Experimental results of the LRGU-MIFS with different sizes of features sets used to train SVM, LR, RF, DT, and LSTM algorithms.

No. of Features	SVM	LR	RF	DT	LSTM
5	0.884	0.881	0.884	0.859	0.884
10	0.92	0.934	0.925	0.943	0.934
15	0.926	0.934	0.925	0.947	0.934
20	0.925	0.934	0.924	0.947	0.934
25	0.927	0.934	0.925	0.946	0.933
30	0.931	0.934	0.924	0.949	0.933
35	0.93	0.934	0.922	0.939	0.933
40	0.93	0.934	0.925	0.947	0.934
45	0.931	0.934	0.925	0.949	0.934

## Data Availability

No new data were created in this study. Data sharing is not applicable to this article.

## References

[1] Ghubaish A., Salman T., Zolanvari M., Unal D., Al-Ali A., Jain R. (2020). Recent Advances in the Internet-of-Medical-Things (IoMT) Systems Security. IEEE Internet Things J..

[2] Ghaleb F.A., Al-Rimy B.A.S., Almalawi A., Ali A.M., Zainal A., Rassam M.A., Shaid S.Z.M., Maarof M.A. (2020). Deep Kalman neuro fuzzy-based adaptive broadcasting scheme for vehicular ad hoc network: A context-aware approach. IEEE Access.

[3] Razdan S., Sharma S. (2022). Internet of medical things (IoMT): Overview, emerging technologies, and case studies. IETE Tech. Rev..

[4] Hasan M.K., Islam S., Memon I., Ismail A.F., Abdullah S., Budati A.K., Nafi N.S. (2022). A Novel Resource Oriented DMA Framework for Internet of Medical Things Devices in 5G Network. IEEE Trans. Ind. Informatics.

[5] Kumar P., Gupta G.P., Tripathi R. (2020). A distributed ensemble design based intrusion detection system using fog computing to protect the internet of things networks. J. Ambient. Intell. Humaniz. Comput..

[6] Rasool R.U., Ahmad H.F., Rafique W., Qayyum A., Qadir J. (2022). Security and privacy of internet of medical things: A contemporary review in the age of surveillance, botnets, and adversarial ML. J. Netw. Comput. Appl..

[7] Hady A.A., Ghubaish A., Salman T., Unal D., Jain R. (2020). Intrusion Detection System for Healthcare Systems Using Medical and Network Data: A Comparison Study. IEEE Access.

[8] Mamdouh M., Awad A.I., Khalaf A.A.M., Hamed H.F.A. (2021). Authentication and Identity Management of IoHT Devices: Achievements, Challenges, and Future Directions. Comput. Secur..

[9] Idrees A.K., Khlief M.S. (2023). Efficient compression technique for reducing transmitted EEG data without loss in IoMT networks based on fog computing. J. Supercomput..

[10] Gupta K., Sharma D.K., Gupta K.D., Kumar A. (2022). A tree classifier based network intrusion detection model for Internet of Medical Things. Comput. Electr. Eng..

[11] Ghazal T.M. (2022). A review on security threats, vulnerabilities, and counter measures of 5G enabled Internet-of-Medical-Things. IET Commun..

[12] Olaimat M.N., Maarof M.A., Al-rimy B.A.S. Ransomware anti-analysis and evasion techniques: A survey and research directions. Proceedings of the 3rd International Cyber Resilience Conference (CRC).

[13] Alghofaili Y., Albattah A., Alrajeh N., Rassam M.A., Al-rimy B.A.S. (2021). Secure Cloud Infrastructure: A Survey on Issues, Current Solutions, and Open Challenges. Appl. Sci..

[14] Khalaf B.A., Mostafa S.A., Mustapha A., Mohammed M.A., Mahmoud M.A., Al-Rimy B.A.S., Abd Razak S., Elhoseny M., Marks A. (2021). An Adaptive Protection of Flooding Attacks Model for Complex Network Environments. Secur. Commun. Netw..

[15] Kashinath S.A., Mostafa S.A., Mustapha A., Mahdin H., Lim D., Mahmoud M.A., Mohammed M.A., Al-Rimy B.A.S., Fudzee M.F.M., Yang T.J. (2021). Review of data fusion methods for real-time and multi-sensor traffic flow analysis. IEEE Access.

[16] Hossen M.N., Panneerselvam V., Koundal D., Ahmed K., Bui F. (2022). M.; Ibrahim, S.M. Federated machine learning for detection of skin diseases and enhancement of internet of medical things (IoMT) security. IEEE J. Biomed. Health Inform..

[17] Urooj U., Maarof M.A.B., Al-rimy B.A.S. A proposed adaptive pre-encryption crypto-ransomware early detection model. Proceedings of the 3rd International Cyber Resilience Conference (CRC).

[18] Al-rimy B.A.S., Maarof M.A., Shaid S.Z.M. (2017). A 0-day aware crypto-ransomware early behavioral detection framework. Recent Trends in Information and Communication Technology.

[19] Papaioannou M., Karageorgou M., Mantas G., Sucasas V., Essop I., Rodriguez J., Lymberopoulos D. (2022). A survey on security threats and countermeasures in internet of medical things (IoMT). Trans. Emerg. Telecommun. Technol..

[20] Sadhu P.K., Yanambaka V.P., Abdelgawad A., Yelamarthi K. (2022). Prospect of internet of medical things: A review on security requirements and solutions. Sensors.

[21] Zaman U., Mehmood F., Iqbal N., Kim J., Ibrahim M. (2022). Towards Secure and Intelligent Internet of Health Things: A Survey of Enabling Technologies and Applications. Electronics.

[22] Sadhu P., Yanambaka V.P., Abdelgawad A., Yelamarthi K. (2022). NAHAP: PUF-based three factor authentication system for internet of medical things. IEEE Consum. Electron. Mag..

[23] Si-Ahmed A., Al-Garadi M.A., Boustia N. (2023). Survey of Machine Learning based intrusion detection methods for Internet of Medical Things. Appl. Soft Comput..

[24] Martins I., Resende J.S., Sousa P.R., Silva S., Antunes L., Gama J. (2022). Host-based IDS: A review and open issues of an anomaly detection system in IoT. Futur. Gener. Comput. Syst..

[25] Rbah Y., Mahfoudi M., Balboul Y., Fattah M., Mazer S., Elbekkali M., Bernoussi B. Machine learning and deep learning methods for intrusion detection systems in iomt: A survey. Proceedings of the 2nd International Conference on Innovative Research in Applied Science, Engineering and Technology (IRASET).

[26] Ashraf E., Areed N., Salem H., Abdelhady E., Farouk A. (2022). IoT Based Intrusion Detection Systems from The Perspective of Machine and Deep Learning: A Survey and Comparative Study. Delta Univ. Sci. J..

[27] Alizadehsani R., Roshanzamir M., Izadi N.H., Gravina R., Kabir H., Nahavandi D., Alinejad-Rokny H., Khosravi A., Acharya U.R., Nahavandi S. (2023). Swarm Intelligence in Internet of Medical Things: A Review. Sensors.

[28] Feng X., Li Q., Wang H., Sun L. Acquisitional rule-based engine for discovering internet-of-things devices. Proceedings of the 27th USENIX Security Symposium.

[29] Saran N., Kesswani N. (2023). A comparative study of supervised Machine Learning classifiers for Intrusion Detection in Internet of Things. Procedia Comput. Sci..

[30] Awotunde J.B., Abiodun K.M., Adeniyi E.A., Folorunso S.O., Jimoh R.G. (2022). A deep learning-based intrusion detection technique for a secured IoMT system. Informatics and Intelligent Applications.

[31] Al-Rimy B.A.S., Maarof M.A., Shaid S.Z.M. (2018). Ransomware threat success factors, taxonomy, and countermeasures: A survey and research directions. Comput. Secur..

[32] Ravi V., Pham T.D., Alazab M. (2022). Attention-Based Multidimensional Deep Learning Approach for Cross-Architecture IoMT Malware Detection and Classification in Healthcare Cyber-Physical Systems. IEEE Trans. Comput. Soc. Syst..

[33] Al-Rimy B.A.S., Maarof M.A., Shaid S.Z.M. (2019). Crypto-ransomware early detection model using novel incremental bagging with enhanced semi-random subspace selection. Futur. Gener. Comput. Syst..

[34] Al-rimy B.A.S., Maarof M.A., Prasetyo Y.A., Shaid S.Z.M., Ariffin A.F.M. (2018). Zero-day aware decision fusion-based model for crypto-ransomware early detection. Int. J. Integr. Eng..

[35] Khalil A.A., E Ibrahim F., Abbass M.Y., Haggag N., Mahrous Y., Sedik A., Elsherbeeny Z., Khalaf A.A., Rihan M., El-Shafai W. (2022). Efficient anomaly detection from medical signals and images with convolutional neural networks for Internet of medical things (IoMT) systems. Int. J. Numer. Methods Biomed. Eng..

[36] Wagan S.A., Koo J., Siddiqui I.F., Qureshi N.M.F., Attique M., Shin D.R. (2023). A fuzzy-based duo-secure multi-modal framework for IoMT anomaly detection. J. King Saud Univ. Comput. Inf. Sci..

[37] Alghanmi N., Alotaibi R., Buhari S.M. (2021). Machine Learning Approaches for Anomaly Detection in IoT: An Overview and Future Research Directions. Wirel. Pers. Commun..

[38] Gökdemir A., Calhan A. (2022). Deep learning and machine learning based anomaly detection in internet of things environments. J. Fac. Eng. Archit. Gazi Univ..

[39] Raj R.J.S., Shobana S.J., Pustokhina I.V., Pustokhin D.A., Gupta D., Shankar K. (2020). Optimal feature selection-based medical image classification using deep learning model in internet of medical things. IEEE Access.

[40] RM S.P., Maddikunta P.K.R., Parimala M., Koppu S., Gadekallu T.R., Chowdhary C.L., Alazab M. (2020). An effective feature engineering for DNN using hybrid PCA-GWO for intrusion detection in IoMT architecture. Comput. Commun..

[41] Chaganti R., Mourade A., Ravi V., Vemprala N., Dua A., Bhushan B. (2022). A Particle Swarm Optimization and Deep Learning Approach for Intrusion Detection System in Internet of Medical Things. Sustainability.

[42] Parimala G., Kayalvizhi R. An effective intrusion detection system for securing IoT using feature selection and deep learning. Proceedings of the International Conference on Computer Communication and Informatics (ICCCI).

[43] Awotunde J.B., Chakraborty C., Adeniyi A.E. (2021). Intrusion Detection in Industrial Internet of Things Network-Based on Deep Learning Model with Rule-Based Feature Selection. Wirel. Commun. Mob. Comput..

[44] Al-Sarem M., Saeed F., Alkhammash E.H., Alghamdi N.S. (2022). An aggregated mutual information based feature selection with machine learning methods for enhancing IoT botnet attack detection. Sensors.

[45] Chaudhary P., Gupta B., Singh A.K. (2022). Implementing attack detection system using filter-based feature selection methods for fog-enabled IoT networks. Telecommun. Syst..

[46] Nimbalkar P., Kshirsagar D. (2021). Feature selection for intrusion detection system in Internet-of-Things (IoT). ICT Express.

[47] Ahmed Y.A., Koçer B., Huda S., Al-rimy B.A.S., Hassan M.M. (2020). A system call refinement-based enhanced Minimum Redundancy Maximum Relevance method for ransomware early detection. J. Netw. Comput. Appl..

[48] Al-Rimy B.A.S., Maarof M.A., Alazab M., Shaid S.Z.M., Ghaleb F. (2021). A.; Almalawi, A.; Ali, A.M.; Al-Hadhrami, T. Redundancy coefficient gradual up-weighting-based mutual information feature selection technique for crypto-ransomware early detection. Future Gener. Comput. Syst..

[49] Kavitha G., Elango N.M. (2021). Genetic Algorithm-Conditional Mutual Information Maximization based feature selection for Bot Attack Classification in IoT devices. J. Mob. Multimedia.

[50] Ahmed Y.A., Huda S., Al-rimy B.A.S., Alharbi N., Saeed F., Ghaleb F.A., Ali I.M. (2022). A weighted minimum redundancy maximum relevance technique for ransomware early detection in industrial IoT. Sustainability.

[51] Elsayed R., Hamada R., Hammoudeh M., Abdalla M., Elsaid S.A. (2022). A Hierarchical Deep Learning-Based Intrusion Detection Architecture for Clustered Internet of Things. J. Sens. Actuator Netw..

[52] Li J., Cheng K., Wang S., Morstatter F., Trevino R.P., Tang J., Liu H. (2017). Feature selection: A data perspective. ACM Comput. Surv..

[53] Qi W., Ovur S.E., Li Z., Marzullo A., Song R. (2021). Multi-Sensor Guided Hand Gesture Recognition for a Teleoperated Robot Using a Recurrent Neural Network. IEEE Robot. Autom. Lett..

[54] Qi W., Aliverti A. (2019). A Multimodal Wearable System for Continuous and Real-Time Breathing Pattern Monitoring During Daily Activity. IEEE J. Biomed. Heal. Inform..

[55] Su H., Qi W., Schmirander Y., Ovur S.E., Cai S., Xiong X. (2022). A human activity-aware shared control solution for medical human–robot interaction. Assem. Autom..

[56] Qi W., Su H. (2022). A Cybertwin Based Multimodal Network for ECG Patterns Monitoring Using Deep Learning. IEEE Trans. Ind. Inform..

[57] Kaushik S., Bhardwaj A., Alomari A., Bharany S., Alsirhani A., Mujib Alshahrani M. (2022). Efficient, Lightweight Cyber Intrusion Detection System for IoT Ecosystems Using MI2G Algorithm. Computers.

